# Tumor-infiltrating CD36^+^CD8^+^T cells determine exhausted tumor microenvironment and correlate with inferior response to chemotherapy in non-small cell lung cancer

**DOI:** 10.1186/s12885-023-10836-z

**Published:** 2023-04-21

**Authors:** Yong-Qiang Ao, Jian Gao, Ling-Xian Zhang, Jie Deng, Shuai Wang, Miao Lin, Hai-Kun Wang, Jian-Yong Ding, Jia-Hao Jiang

**Affiliations:** 1 Department of Thoracic Surgery, Zhongshan Hospital, Fudan University, 180 Fenglin Road, Shanghai, 200032 P. R. China; 2 Cancer Center, Zhongshan Hospital, Fudan University, Shanghai, China; 3 Department of Cardiothoracic Surgery, the Second Affiliated Hospital of Nanchang University, Nanchang, 330000 P.R. China; 4 Institute of Vascular Disease, Shanghai TCM-Integrated Hospital, Shanghai University of Traditional Chinese Medicine, Shanghai, China; 5 CAS Key Laboratory of Molecular Virology and Immunology, Institute Pasteur of Shanghai, Chinese Academy of Sciences, Shanghai, China

**Keywords:** NSCLC, CD36^+^CD8^+^ T cells, Tumor microenvironment, Prognosis, Chemotherapy

## Abstract

**Background:**

The scavenger receptor CD36 was reported to be highly expressed on tumor-infiltrating CD8^+^ T cells, but the clinical role remains obscure. This study aims to explore the infiltration and clinical value of CD36^+^CD8^+^ T cells in NSCLC.

**Methods:**

Immunohistochemistry and immunofluorescence were conducted for survival analyses and immunological evaluation in 232 NSCLC patients in Zhongshan Hospital. Flow cytometry analyses were carried out to assess the immune cells from fresh tumor samples, non-tumor tissues and peripheral blood. In vitro tumor infiltrating lymphocytes cultures were conducted to test the effect of CD36 blockage.

**Results:**

Accumulation of CD36^+^CD8^+^ T cells in tumor tissues was correlated with more advanced stage (p < 0.001), larger tumor size (p < 0.01), and lymph node metastasis (p < 0.0001) in NSCLC. Moreover, high infiltration of CD36^+^CD8^+^ T cells indicated poor prognosis in terms of both overall survival (OS) and recurrence-free survival (RFS) and inferior chemotherapy response. CD36^+^CD8^+^ T cells showed decreased GZMB (p < 0.0001) and IFN-γ (p < 0.001) with elevated PD-1 (p < 0.0001) and TIGIT (p < 0.0001). Analysis of tumor-infiltrating immune cell landscape revealed a positive correlation between CD36^+^CD8^+^ T cells and Tregs (p < 0.01) and M2-polarized macrophages (p < 0.01) but a negative correlation with Th1 (p < 0.05). Notably, inhibition of CD36 partially restored the cytotoxic function of CD8^+^ T cells by producing more GZMB and IFN-γ.

**Conclusion:**

CD36^+^CD8^+^ T cells exhibit impaired immune function and high infiltration of CD36^+^CD8^+^ T cells indicated poor prognosis and inferior chemotherapy response in NSCLC patients. CD36 could be a therapeutic target in combination with chemotherapy in NSCLC patients.

**Supplementary Information:**

The online version contains supplementary material available at 10.1186/s12885-023-10836-z.

## Background

Non-small cell lung cancer is the primary cause of cancer-related death worldwide [1]. Early-stage or localized NSCLC is treatable with surgical interventions, with favorable 5-year overall survival in most postoperative patients [2]. Nevertheless, patients with lymph node metastasis or at advanced stage have poor prognosis [3]. According to the National Comprehensive Cancer Network guidelines, platinum-based postoperative chemotherapy is currently the first-line treatment for operable patients [4]. However, over 70% of patients relapse and expire, mainly because of impaired anti-tumor immune response and tumor metastatic progression [5]. To reveal the potential correlation between immunosuppression with inferior response to chemotherapy in NSCLC, many studies shifted the focus from NSCLC cells to the surrounding microenvironment through single cell sequencing. Researchers have demonstrated that CD8^+^ T cells are enriched in the TME of NSCLC, but its correlation with clinical prognosis and chemotherapy response is still understudied [6]. Moreover, a suppressed immune microenvironment in advanced NSCLC were also reported [7]. The reversion of CD8^+^ T cells which were suppressed by specific exhausted markers or tumor cells is now an urgent issue to be addressed and has promising prospects for NSCLC treatment.

CD8^+^ T cells can be transformed from the initial effector state to the dysfunctional state with two features: decreased cytotoxic cytokines and sustained expression of multiple inhibitory receptors, which co-determine the exhaustion of CD8^+^ T cell in the TME. CD36, a scavenger receptor expressed in multiple cell types in TME [8], especially effector T cells, Tregs, dendritic cells and myeloid cells, has been demonstrated to be involved in regulating anti-tumor immune response through different mechanisms [9]. CD8^+^ T cells, which play a major role in anti-tumor immunity, have been confirmed to express CD36 via single cell sequencing [10]. The mechanisms by which CD36 affects the immune function of CD8^+^ T cells have been partially demonstrated, including ferroptosis and dysfunction caused by lipid peroxidation [8, 10, 11]. In summary, the clarified role of CD36^+^ CD8^+^ T cells may be the key to predict the response to targeted therapy and prognosis of tumor patients.

**Fig. 1:**
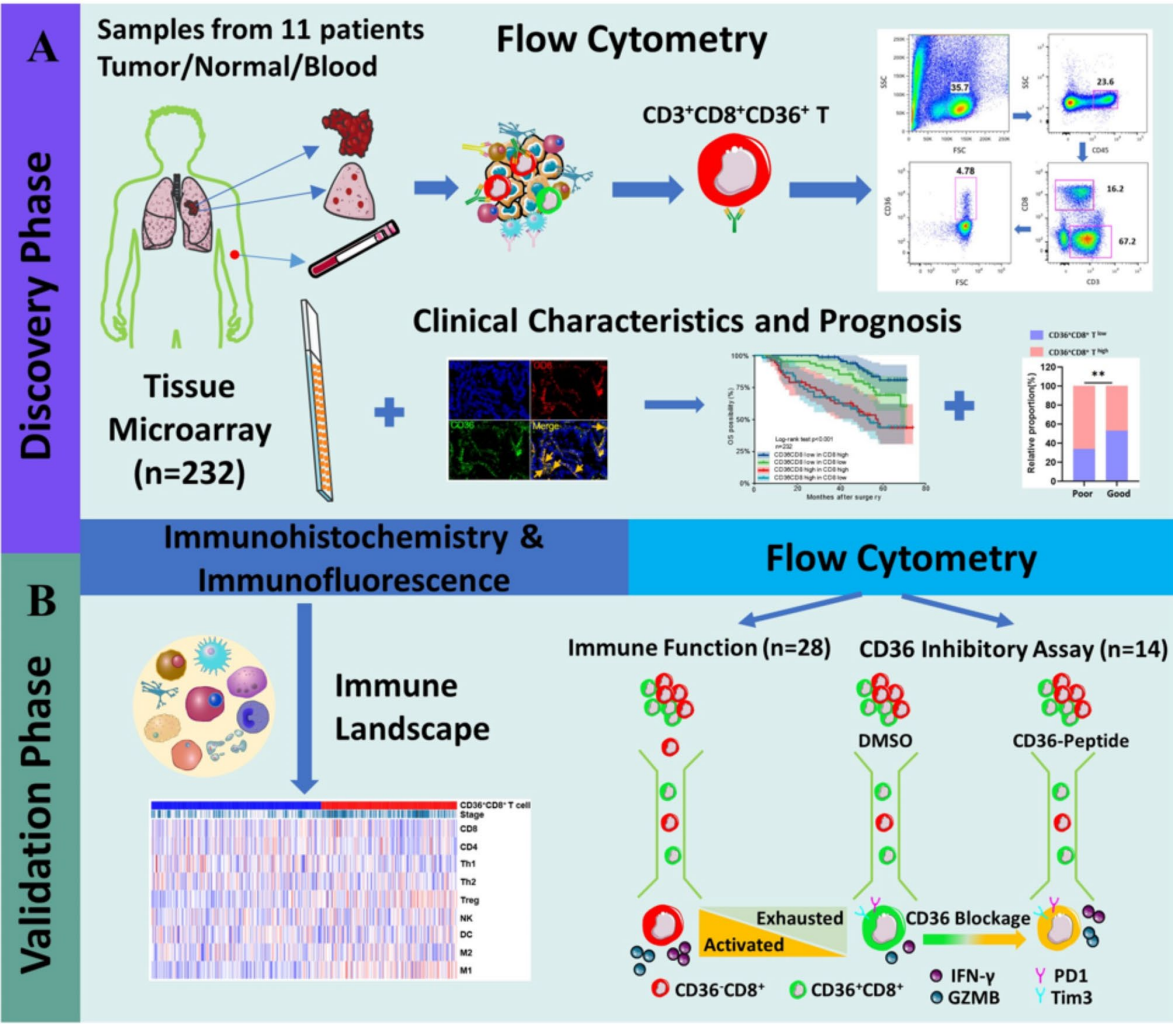
Methodology and experimental overview. **(A)** We firstly found a significant higher proportion of CD36^+^CD8^+^ T cells in tumors compared with nontumor tissues and PBMCs via flowcytometry in 11 NSCLC patients. Then dual IF was conducted in tissue microarray and revealed that CD36^+^CD8^+^ T cells infiltration correlated with worse patients’ clinicopathological characteristics and prognosis. **(B)** Based on these findings, we further explored the mechanisms how CD36 was involved in affecting CD8^+^ T cells function and reshaped TME of NSCLC. In addition, the effects of CD36^+^CD8^+^ T cells on chemotherapy and the feasibility of blocking CD36 to restore the function of CD8^+^ T cells were further explored

However, the function of CD36^+^ CD8^+^ T cells in TME has not been fully demonstrated. To our knowledge, no study has focused on the correlation of CD36^+^CD8^+^ T cells with the prognosis and response to chemotherapy in NSCLC patients. In this study, flow cytometry and immunofluorescence were used to demonstrate the high expression of CD36 on CD8^+^ T cells, indicating exhausted immune function, with a correlation to worse clinical characteristics, worse prognosis, and inferior chemotherapy response in NSCLC patients. Considering this, CD36-inhibitor (Sulfosuccinimidyl oleate (SSO), MCE, Shanghai, China) was used and the restored function of CD8^+^ T cells was observed. Figure 1 summarizes the methodology and experimental overview of this research. Our results suggested that CD36 could be a potential immune checkpoint to recover the immune response against tumor, and CD36 blockage combined with chemotherapy would improve the survival of NSCLC patients.

## Materials and methods

### Patients and clinical database

This study was approved by the Research Ethics Committees of Zhongshan Hospital, Fudan university, Shanghai, China. A total of 232 NSCLC patients (age range 32–78 years, median 57 years) with localized NSCLC who accepted surgical treatment at the Department of Thoracic Surgery, Zhongshan Hospital, Fudan University were enrolled in this study. All patients were followed up every 6 months, and the last follow-up was on September 20th, 2021. Patients at or over TNM stage IIA have received postoperative adjuvant chemotherapy. Patient inclusion criteria include: informed consent; NSCLC with surgery and confirmed pathological result; no history of other malignancies. Exclusion criteria include: lack of tissue sample; necrosis area greater than 80% in tumor; preoperative systemic therapy including neoadjuvant chemotherapy. Patients and their families were fully informed before the operation that the surgical samples would only be used for scientific research and the operation would not be affected. Clinicopathological characteristics of NSCLC patients are exhibited in Supplementary Table 1.

The baseline demographic and clinical data including chemotherapy and survival information were collected retrospectively from electronic medical records and follow-up. Tumor stage and lymph node metastasis were resigned based on the 8th AJCC TNM classification for NSCLC postoperatively. Tumor sizes were diagnosed based on radiographic evidence and postoperative pathological results. Disease progression was defined according to the RECIST1.1 criteria. All pathological results were reviewed by two experienced pathologists independently, and a third pathologist will give the final diagnosis upon any controversy between the previous observers. Pathological results including histological type, differentiation, tumor size, invasion, lymph node metastasis, epidermal growth factor receptor (EGFR) gene mutation status and programmed cell death ligand 1 (PD-L1) expression were analyzed. In order to accurately explore the infiltration of immune cells in the tumor microenvironment, a total of 53 fresh NSCLC tissue samples were randomly selected during the operation for further analysis by flow cytometry. Clinicopathological characteristics of the sampled patients are exhibited in Supplementary Table 2.

### Immunofluorescence (IF) and immunohistochemistry (IHC) assay

To confirm the co-expression of CD36 and CD8 on tumor infiltrating T lymphocytes, dual immunofluorescence was performed first, and the infiltration of other immune cells was explored by immunohistochemistry later. Tissue microarray construction and the IHC protocol have been described previously [12]. The details of the antibodies used in IHC assay are provided in Supplementary Table 3. IHC sections were scanned by Olympus CDD camera, Nikon eclipse Ti-S microscope (200×magnification) and NIS-Elements F3.2 software. Two experienced pathologists, blinded to the follow-up information, counted the number of positive staining cells at 200× magnification, and the average number was used as the final data. For IF staining, the slides were incubated with specific antibodies at 4 °C overnight. Then, samples were incubated with species-appropriate rabbit/mouse secondary antibodies coupled to Alexa Fluor dyes (488 (ab185033),594 (ab203419)) for 1 h at room temperature. DAPI (ab285390) containing anti-fluorescence quenching was used to mount cover slips, and the sections were analyzed through Leica DMi8 microsystems.

### Flow cytometry

The details of flow cytometry have been described previously [13]. A total of 53 resected fresh specimen from NSCLC patients were randomly collected for flow cytometry analyses. The first 11 specimen and corresponding non-tumor tissues and peripheral blood were used to explore distribution differences of CD36^+^CD8^+^ T cells among tumors, non-tumor tissues and peripheral blood. Another 28 specimen were used to analyze the immune function of CD36^+^CD8^+^ T cells and the last 14 specimen were used to examine the effect of CD36 inhibition. Peripheral blood mononuclear cells (PBMCs) were isolated from heparinized venous blood by lysing solution (BD Biosciences). Fresh tumor and non-tumor tissues (at least 2 cm away from the tumor site) were minced, ground and digested with collagenase IV in tissue suspension preparation. After that, 70 μm cell strainers were used to filter and collect single cell suspensions. Then, PBMCs and single cells were stained with appropriate monoclonal antibodies for 30 min at 4 °C. Intracellular cytokines were stimulated with phorbol myristate acetate (PMA, 10 ng ml^− 1^, Sigma) and ionomycin (0.5 µg ml^− 1^, Sigma) for 4 h at 37 °C in RPMI-1640 medium containing 10% FBS, and 5 µg ml^− 1^ brefeldin A (BFA, Sigma) was added 2 h before the end of stimulation. Stimulated cells were collected and blocked with Fc-blocker and 10% fetal serum for 5 min on ice, followed by staining with surface-marker antibodies for 30 min on ice. Cell suspensions were then washed with PBS, fixed with 3.7% paraformaldehyde (15 min, room temperature), and permeabilized with 0.2% saponin (15 min on ice). Cell suspensions were lastly stained with specific fluorochrome-labeled antibodies for 30 min at 4 °C. The antibodies used and the gating strategy are provided in Supplementary Tables 4 and Supplementary Fig. 1, respectively. Flow cytometry was performed with a BD FACS Fortessa (Beckman Coulter, Brea, California, USA), and cells were analyzed using FlowJo software V.10 (Treestar, San Carlos, California, USA).

### In vitro CD36 inhibition assay

Fresh NSCLC tissue was washed twice with RPMI-1640 medium containing 10% fetal bovine serum before being minced. The specimens were stored in RPMI-1640 medium containing 1 mg/ml collagenase IV and incubated at 37 °C for 3 h with continuous rotation, and the cell suspension was later filtered through a 70 μm cell strainer (BD Biosciences). The collected cells were cultured in RPMI-1640 containing 10% fetal bovine serum and 300ul/ml IL-2 (in plates coated with 10ug/ml CD3 antibody and CD28 antibody), and SSO (50 µM/L) or vehicle (DMSO) were added respectively. After 24 h culture at 37 °C and 5% CO_2_ overnight, cells were collected and analyzed by flow cytometry as above.

### Statistical analysis

Categorical variables were compared by Chi-square test and Fisher’s exact test to assess the correlations between CD36 expression and patient characteristics. Continuous variables were compared by parametric (Student’s t test, paired t test and Pearson’s test) or nonparametric (Mann–Whitney U test and Spearman’s test) tests for correlation analysis and evaluation on differences in mean ± SD. Kaplan–Meier method and log-rank test were used to demonstrate survival curves between different groups. Overall survival (OS) and recurrence-free survival (RFS) were calculated from the date of surgery to time of death or recurrence, respectively. COX multivariable regression analysis was used to explore factors affecting OS and RFS rate with R package survival. We used the average (ratio of CD36^+^CD8^+^/CD8^+^ T cells = 0.322) as the cut-off in the definition of CD36^+^CD8^+^ high and low group. All analyses were two-tailed and performed at a significance level of 5% (*p* < 0.05) using SPSS version 21.0 (For Windows; Chicago, IL, USA), RStudio V.3.5.5 (Boston, Massachusetts, USA) with additional Bioconductor packages and GraphPad Prism V.7.0 (La Jolla, California, USA).

## Results

### The relationship of CD36^+^CD8^+^T cells level and clinical characteristics of NSCLC

**Fig. 2:**
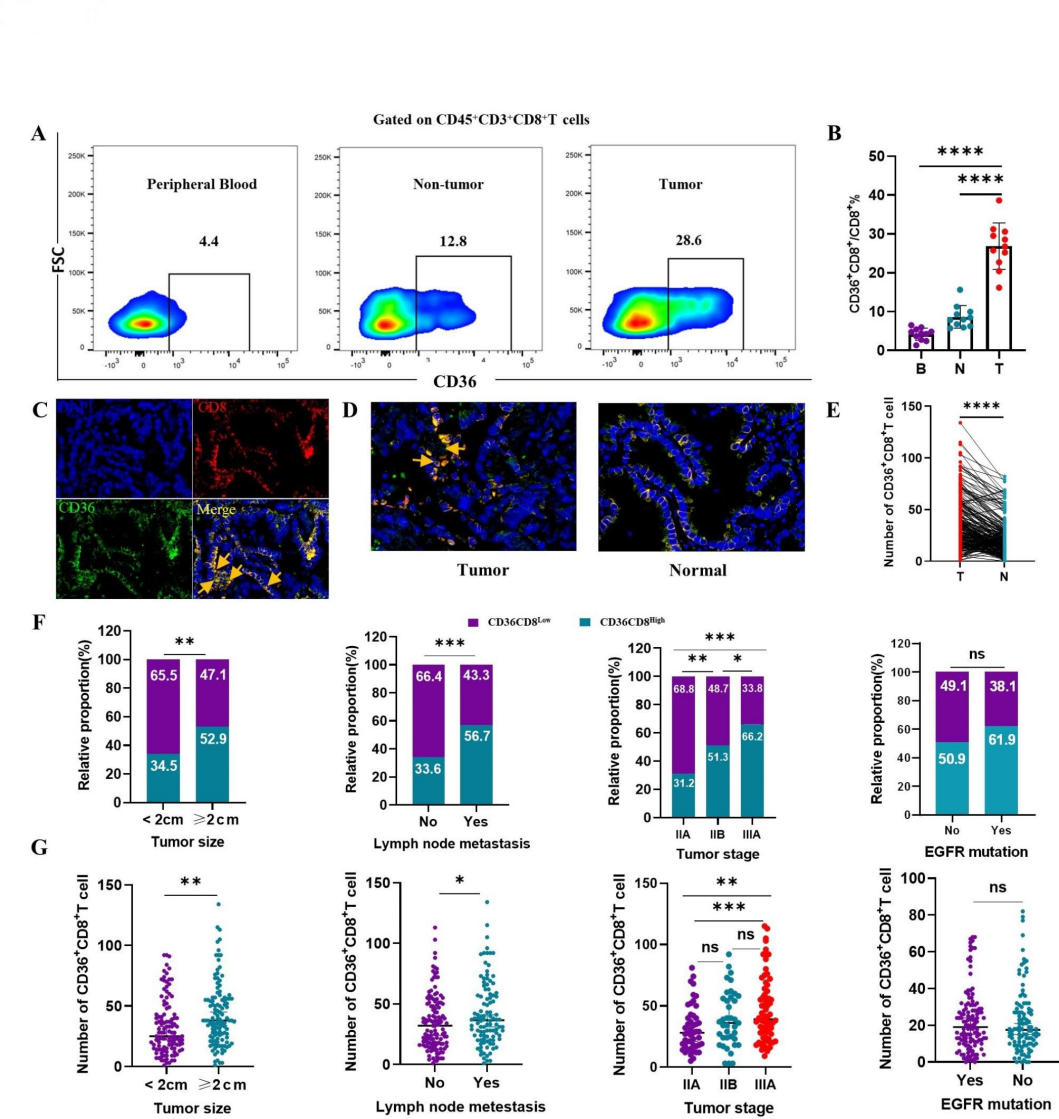
Accumulation of CD36^+^CD8^+^ T cells in NSCLC is correlated with disease progression. **A, B.** The representative flow cytometry and statistics analysis of CD36 expression on CD8^+^ T cells in matched PBMCs, non-tumor tissues and tumor from patients with NSCLC (n = 11, ****p < 0.0001 by paired t test). **C.** Representative images of the immunofluorescence staining with DAPI (blue), CD36 (green), CD8 (red), and merge (double positive) on NSCLC tissues. Scans were imaged at 200 magnifications. **D, E.** The proportion of CD36^+^CD8^+^ T cells is higher in tumor than that in non-tumor tissues (****p < 0.0001 by Student’ s t test). **F, G.** The number and proportion of CD36^+^CD8^+^ T cells was elevated in patients with larger tumor size (**p < 0.01), lymph node metastasis (***p < 0.0001) and advanced TNM stage (***p < 0.001). However, in patients with or without EGFR mutation, the number and proportion of CD36^+^CD8^+^ T cells was not statistically different (Chi square test and paired t test, respectively). B = blood, N = non-tumor, T = tumor

We examined the number of CD36^+^CD8^+^ T cells in tumors, non-tumor tissues and PBMCs taken from 11 NSCLC patients. Compared with PBMCs and non-tumor tissues, the infiltration of CD36^+^CD8^+^ T cells was higher in tumor (*p* < 0.0001, Fig. 2A and B). To further confirm this finding, we stained fixed sections and tumor micro-array (TMA) for CD8 and CD36 by immunofluorescence in NSCLC patients, and more CD36^+^CD8^+^ T cells in tumors were found compared with non-tumor tissues (*p* < 0.0001, Fig. 2C-E). In addition, we noticed that the expression of CD36 in CD8^+^ T cells was higher than tumor cells (Fig. 2C and D). Notably, a significant increase in both the number and proportion of CD36^+^CD8^+^ T cells in patients with more advanced stage (*p* < 0.001), larger tumor size (*p* < 0.01), and lymph node metastasis (*p* < 0.0001) was observed, but the number and proportion of CD36^+^CD8^+^ T cells in patents with or without EGFR mutation had no significant difference (Fig. 2F and G).

### Accumulation of CD36^+^CD8^+^T cells in NSCLC predicted poor prognosis

**Fig. 3:**
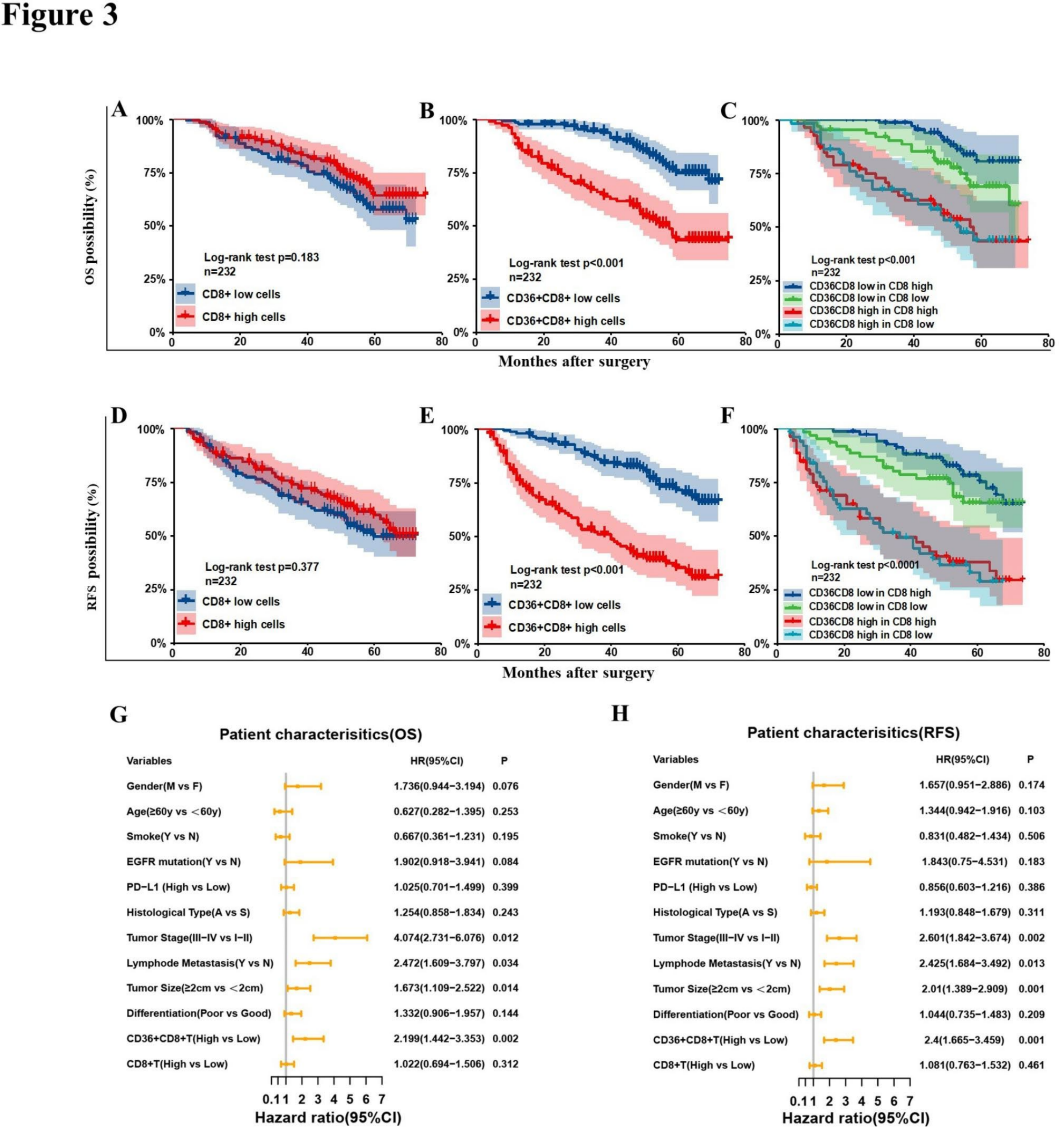
Infiltration of CD36^+^CD8^+^ T cells predict poor prognosis. Kaplan–Meier curve of overall survival (OS) and recurrence-free survival (RFS) of 232 NSCLC patients according to the CD8^+^ T cells counts **(A, D)**, proportion of CD36^+^CD8^+^ cells **(B, E)** and the combination of both markers **(C, F)**. Log-rank tests were used to derive *p* values for comparisons between groups. **G, H.** Multivariate Cox analysis identified independent prognostic factors for OS and RFS in NSCLC patients. HR, hazard radio; CI, confidence interval

The Kaplan–Meier curve showed that there was no significant difference in OS (*p* = 0.183) and RFS (*p* = 0.377) between patients with high and low CD8^+^ T cells (Fig. 3A and D). However, we found that accumulation of CD36^+^CD8^+^T cells was correlated with worse prognosis in terms of both OS (*p* < 0.001) and RFS (*p* < 0.001, Fig. 3B and E). Thus, we further analyzed the impact of CD36^+^CD8^+^T cells on the survival of NSCLC patients with high or low CD8^+^T cell infiltration. It turned out that in patients with high CD8^+^T cells infiltration, the subgroup with higher proportion of CD36^+^CD8^+^ cells reported worse OS compared to those that with lower proportion (*p* < 0.001, Fig. 3C), and similar trend was shown in the RFS comparison (*p* < 0.001, Fig. 3F). Multivariable analysis showed that besides tumor size and tumor invasion, the ratio of CD36^+^CD8^+^T cells was also an independent risk factor for OS (*p* = 0.005, Fig. 3G) and RFS (*p* < 0.000, Fig. 3H).

### Infiltration of CD36^+^CD8^+^ T cells is associated with decreased cytotoxic cytokine production

**Fig. 4:**
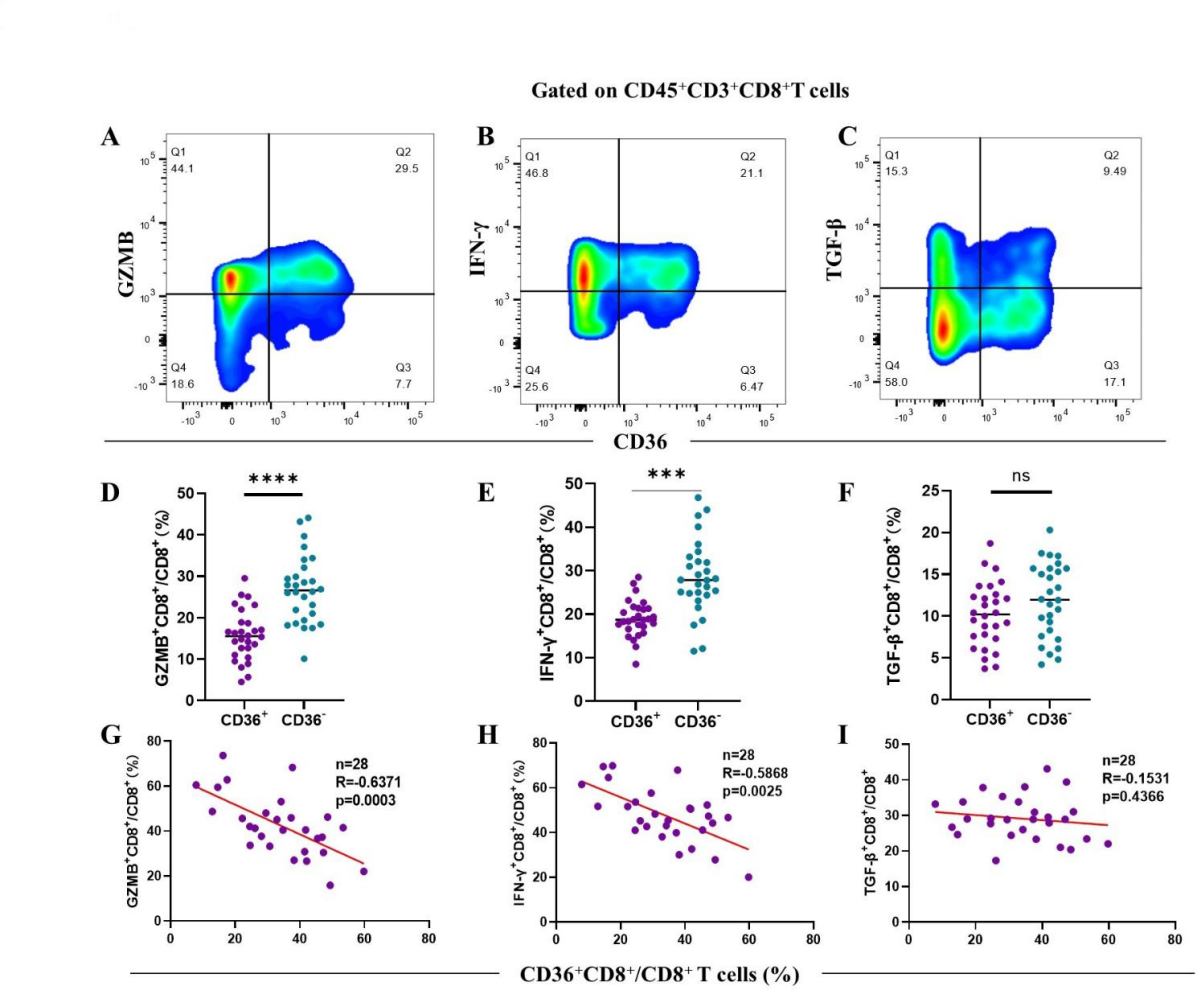
The function of CD36^+^CD8^+^ T cells are impaired. **A-C.** Representative flow cytometry plots for GZMB, IFN-γ and TGF-β in CD36-positive and CD36-negative CD8^+^ T cells with quantification below of 28 patients **(D-F)**. **G-I.** Correlation assessed by Pearson’s correlation analysis and linear regression analysis between proportion of CD36^+^CD8^+^ T cells and the frequencies of three effector markers in NSCLC patients based on the results of flow cytometry. *p < 0.05; ***p < 0.001; ****p < 0.0001 by paired t test

Next, we explored the immune function of CD36^+^CD8^+^ T cells in 28 fresh NSCLC tissues by flow cytometry. The proportion of GZMB^+^ T cells (*p* < 0.0001) and IFN-γ^+^ T cells (*p* < 0.001) was reduced in the CD36^+^CD8^+^ T cells subgroup (Fig. 4A, B, D and E). Moreover, our measurement showed that CD36^+^CD8^+^ T cells infiltration was negatively correlated with GZMB^+^ CD8^+^ T cell (R = -0.6340, *p* = 0.0003) and IFN-γ^+^CD8^+^ T cells (R=-0.5483, *p* = 0.0025) (Fig. 4G H). Although we observed no difference of TGF-β expression between CD36^+^CD8^+^ T cells and CD36^–^CD8^+^ T cells (Fig. 4C F and 4I), these findings indicated that the cytotoxic function of CD36^+^CD8^+^ T cells were impaired in the TME of NSCLC.

### Increased immune checkpoint expression is correlated with infiltration of CD36^+^CD8^+^ T cells

**Fig. 5:**
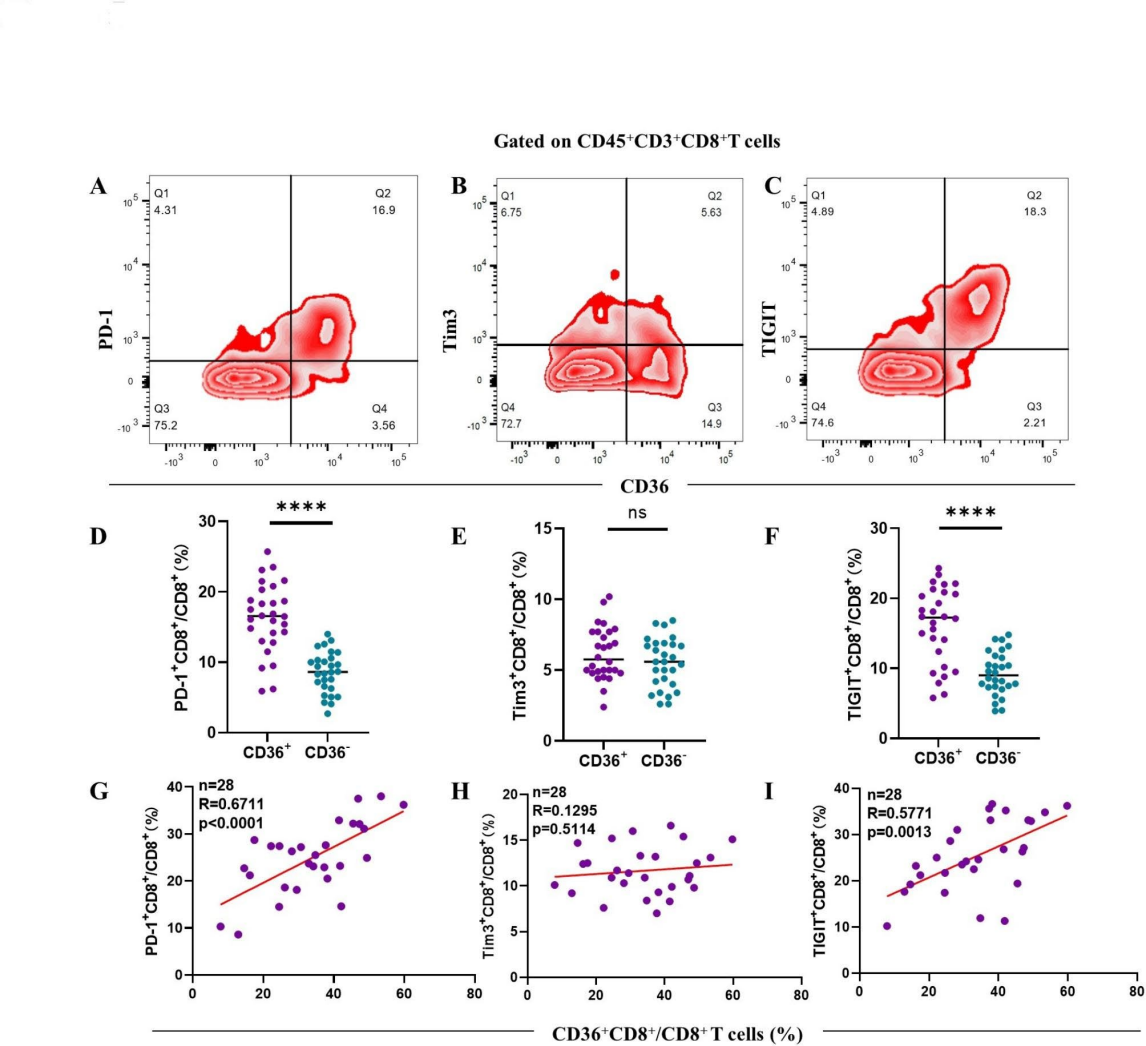
CD36^+^CD8^+^ T cells exhibit immunosuppressive state. **A, C.** The expression of PD-1 and TIGIT are increased on the CD36-positive CD8^+^ T cells () compared with CD36-negative CD8^+^ T cells by paired t test **(D, F)** and correlation analysis **(G, I)** (n = 28) while no significant difference of Tim3 expression between CD36^+^CD8^+^ T cells and CD36^–^CD8^+^ T cells was observed **(B, E, H)**. *p < 0.05; ****p < 0.0001 by paired t test

Except for cytotoxic function mediated by pro-inflammatory cytokines, the immune checkpoints including PD-1, TIM-3 and TIGIT expressed on CD36^+^CD8^+^ T cells were also analyzed. Compared with CD36-negative CD8^+^ T cells, CD36-positive CD8^+^ T cells expressed higher level of PD-1 (*p* < 0.0001) and TIGIT (*p* < 0.0001, Fig. 5A and D C, 5 F). In addition, we observed a positive correlation between infiltration of CD36^+^CD8^+^ T cells and both PD-1 (R = 0.6711, *p* < 0.0001) and TIGIT expression (R = 0.5771, *p* = 0.0013, Fig. 5G and I), indicating up-regulated immune checkpoint expression and compromised immune function of CD36^+^CD8^+^ T cells. However, the expression of Tim3 between CD36^+^CD8^+^ T cells and CD36^–^CD8^+^ T cells had no statistical difference (Fig. 5B and E H).

### Infiltration of CD36^+^CD8^+^ T cells is accompanied by Tregs and M2 in the TME

**Fig. 6:**
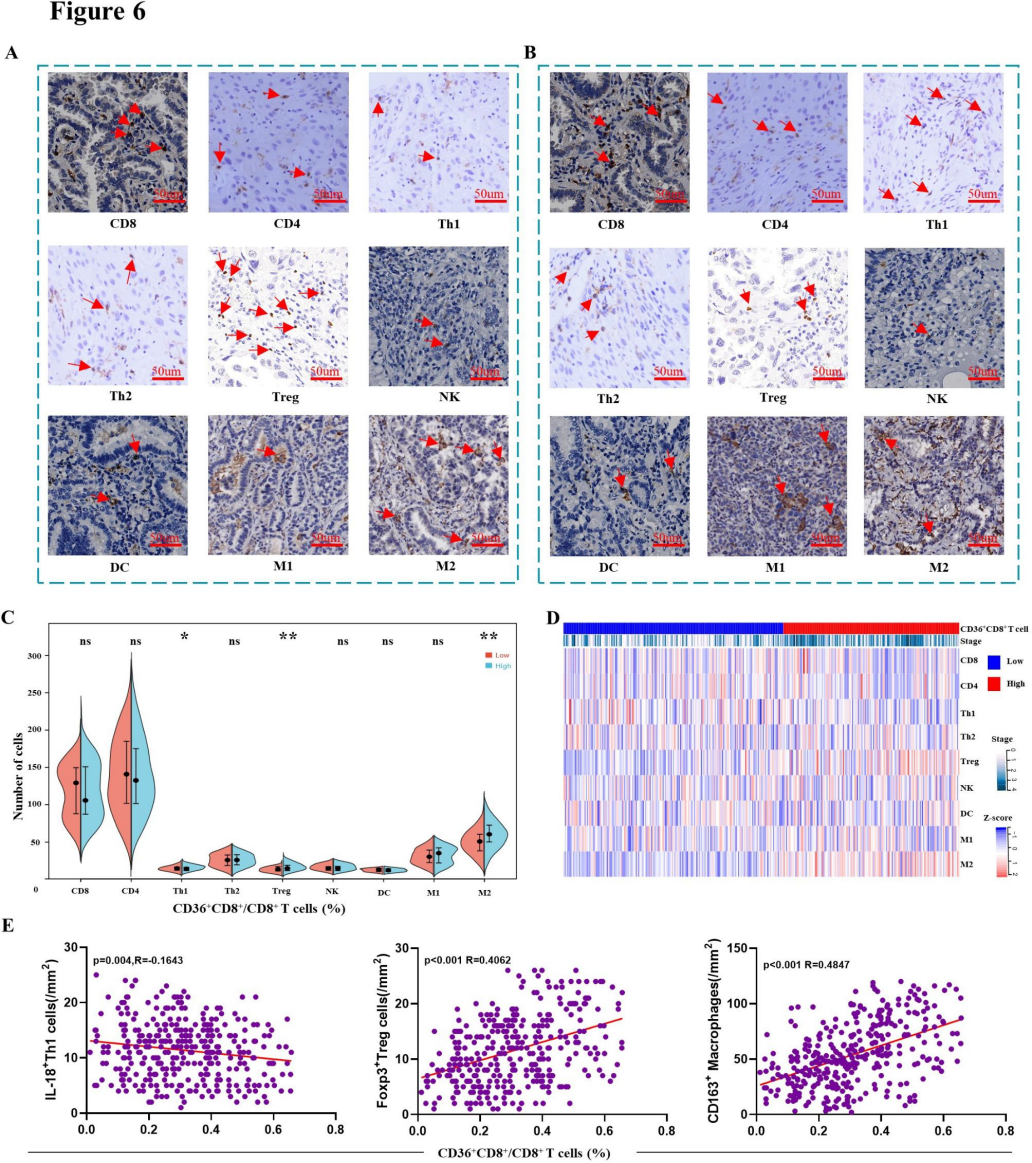
Infiltration of CD36^+^CD8^+^ T cells in the TME is accompanied by Tregs and M2. **A, B.** Representative pictures of immune cell infiltration in CD36^+^ T cells high or low group. **C.** Comparison of various immune cell types between high and low CD36^+^CD8^+^ T cells proportion subgroups. **D.** Heat map displays scaled expression of various immune cell types between high and low CD36^+^CD8^+^ T cells proportion subgroups. **E.** Linear regression showed a positive correlation between CD36^+^CD8^+^ T cells with Tregs or M2. ns: no significance, *p < 0.05 and **p < 0.01 by Mann–Whitney U test; CD8 = CD8^+^ T cells; CD4 = CD4^+^ T cells; Th1 = Type 1 T helper cells; Th2 = Type 2 T helper cells; Treg = regulatory T cells; NK = nature killer cells; DC = dendritic cells; M1 = M1 macrophages; M2 = M2 macrophages

CD8^+^ T cells are often regulated by other immune cells, so we further explored the correlation between the infiltration of CD36^+^CD8^+^ T cells and the entire immune landscape in NSCLC. A total of 9 types of immune cells were employed to define the immune landscape, and high infiltration of CD36^+^CD8^+^ T cells was found to be accompanied by more immunosuppressive cells including Tregs (*p* < 0.01) and M2 (*p* < 0.01, Fig. 6A-D). Moreover, a decrease of Th1 cells in higher CD36^+^CD8^+^ T cells infiltration group (*p* < 0.05) was also observed. As for other immune cells, no significant difference was found between high and low CD36^+^CD8^+^ T cells groups. Correlation analysis was performed subsequently, and results showed that higher proportion of CD36^+^CD8^+^ T cells was associated with more Treg (R = 0.4062, *p* < 0.001) and M2 (R = 0.4847, *p* < 0.001) infiltration, but not Th1 infiltration (R= -0.1643, *p* = 0.004, Fig. 6E).

### High proportion of CD36^+^CD8^+^T cells correlated with inferior response to chemotherapy

**Fig. 7:**
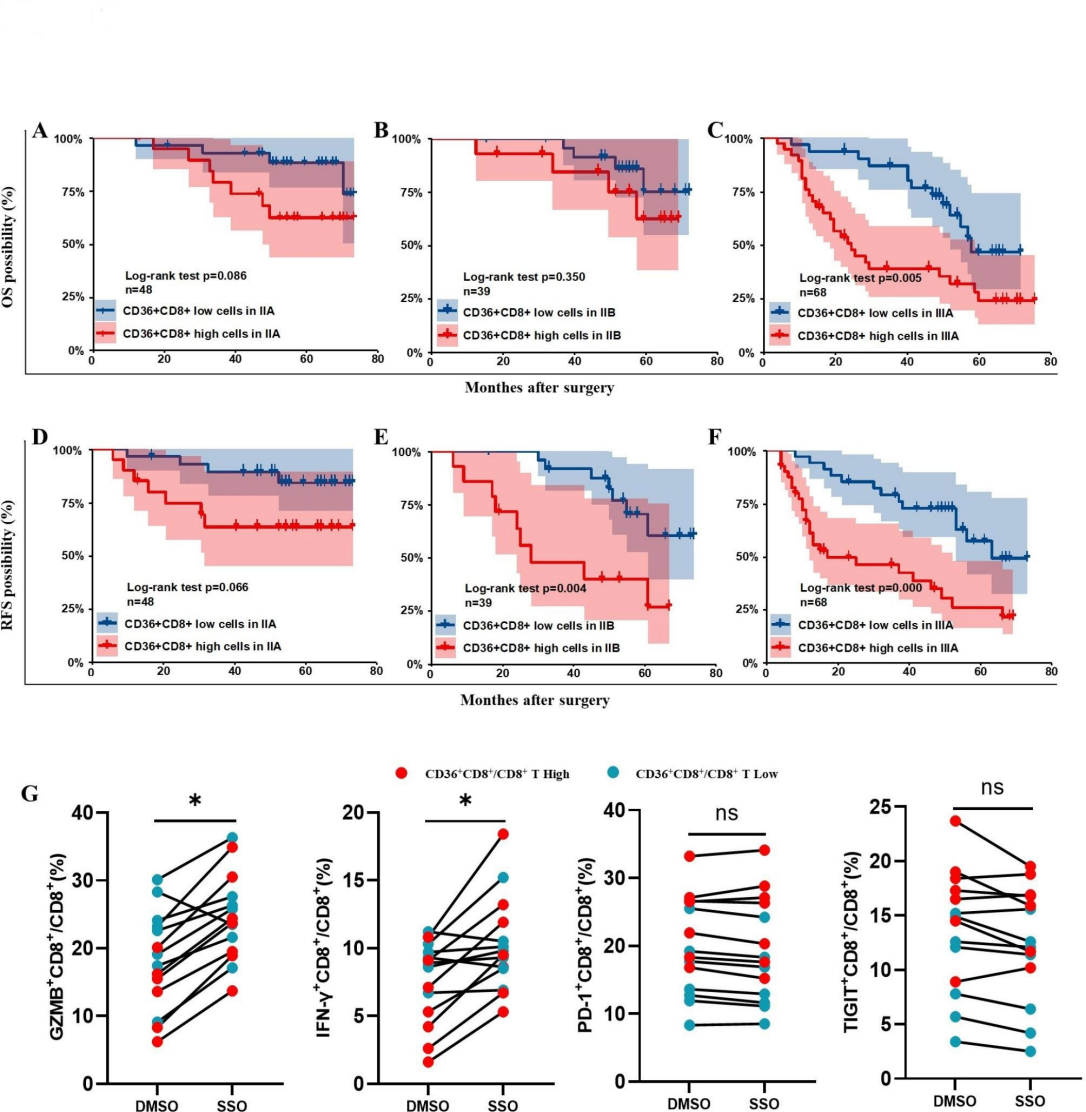
CD36^+^CD8^+^ T cells predict poor therapeutic response to chemotherapy. **A, B, D.** NSCLC patients with low infiltration of CD36^+^CD8^+^ T cells at early stages showed no significant difference in chemotherapy response and prognosis compared to patients with high infiltration of CD36^+^CD8^+^ T cells. **C, E, F.** Patients with high infiltration of CD36^+^CD8^+^ T cells at advanced stages showed significant difference in chemotherapy response and prognosis compared to patients with low infiltration of CD36^+^CD8^+^ T cells. **G.** Level changes of GZMB^+^, IFN-γ^+^, PD-1^+^ and TIGIT^+^ CD8^+^ T cells from patients (n = 14) after SSO or DMSO treatment. ns: no significance, *p < 0.05 by paired t test

The infiltration of CD36^+^CD8^+^ T cells was positively correlated with TNM stage of NSCLC patients (Fig. 2F and G). Considering that patients with advanced TNM stage (IIA-IIIA) usually receive adjuvant chemotherapy after surgery in clinical practice, OS and RFS of patients receiving postoperative chemotherapy, under the condition of both high and low CD36^+^CD8^+^T infiltration, were also analyzed. The Kaplan–Meier curve showed that accumulation of CD36^+^CD8^+^T cells was correlated with worse prognosis in terms of both OS (*p* = 0.005) and RFS (*p* = 0.000) in stage IIIA, and RFS (*p* = 0.004) in stage IIB (Fig. 7C and E F), while no significant difference of both OS and RFS was observed in patients at stage IIA and OS in patients at stage IIB (Fig. 7A, B and D). These data showed that proportion of CD36^+^CD8^+^ T cells may negatively affect survival prognosis of NSCLC patients administered with postoperative chemotherapy.

### Immune function of CD36^+^CD8^+^ T cells can be partially restored by CD36 inhibitor

Given the potential impact on the TME of NSCLC and clinical significance, we tried to test the therapeutic effect of targeting CD36. The isolated immune cells from fresh NCSLC tissue were cultured with SSO (50 µM/L) or vehicle for 24 h. The cells were then collected and examined for cytotoxic function and immune checkpoint expression through flow cytometry. The proportion of GZMB^+^ cells (*p* < 0.05) and IFN-γ^+^ (*p* < 0.05) was recovered after SSO treatment, especially in the subgroup with high proportion of CD36^+^CD8^+^ T cells (Fig. 7G). However, SSO didn’t change the expression level of immune checkpoints of CD36^+^CD8^+^ T cells. These results indicated that CD36-targeted therapy could enhance antitumor immune response.

## Discussion

The NSCLC TME consists of tumor cells and stromal cells, including distinct immune cell subsets. CD36, a scavenger receptor expressed in multiple cell types mediating lipid uptake and immunological recognition, plays an important role in TME remodeling. In this study, we discovered the suppressive function of CD36^+^CD8^+^ T cells in NSCLC and demonstrated a higher proportion of CD36^+^CD8^+^ T cells in NSCLC tissues than that in non-tumor tissues, which was positively correlated with TNM stage. From the best of our knowledge, the role of CD36 in NSCLC cells proliferation and migration [14], and its function in macrophage polarization during acute lung injury [15] had been demonstrated. However, no research was conducted in NSCLC to explore the expression and distribution of CD36 in tumor-infiltrating T lymphocytes. And the mechanism of CD36^+^CD8^+^ T cells leading to tumor progression was not clear. Some recent studies mainly focused on the role of CD36 in regulating cholesterol or fatty acids metabolism and subsequent ferroptosis [11, 16, 17]. Our research in NSCLC showed that anti-tumor function of CD36^+^CD8^+^ T cells was impaired, with less production of GZMB and INF-γ and higher expression of PD-1 and TIGIT. The decreased cytotoxicity and increased suppressive molecules contribute to tumor growth and evasion, which result in the poor outcomes of NSCLC patients. More importantly, a negative correlation between cytotoxic cytokines and CD36^+^CD8^+^ T cells infiltration was observed, as well as a positive correlation between CD36^+^CD8^+^ T cells infiltration and the expression of immune checkpoints. Further exploration found that suppressive immune components increased in the TME with high proportion of CD36^+^CD8^+^ T cells. More immunosuppressive cells (Tregs and M2) were observed in high CD36^+^CD8^+^ T cells group. Except for Th1 cells, main effector cells (CD4^+^ T, NK, M1, DC cells) showed no differences between high and low CD36^+^CD8^+^ T cells infiltration groups. Additionally, the proportion of CD36^+^CD8^+^ T cells was positively correlated with the infiltration of Treg and M2 cells. The mechanisms of increased Treg and M2 cells infiltration in the TME had been explored [18, 19], but no evidence confirmed their relationship with the increase of CD36^+^CD8^+^ T cells. Nevertheless, since Treg and M2 cells can directly inhibit the function of CD8^+^ T cells, and Treg cells can selectively sustain M2-like macrophage metabolic fitness [20], the high expression of CD36 and the high infiltration of Treg and M2 cells establish a vicious circle of immunosuppression, which together impair the function of CD8^+^ T cells, promoting tumor immune escape and poor prognosis of NSCLC patients. CD8^+^ T cells have long been thought to exert anti-tumor effects in different solid tumors including lung cancer. However, a study from Mori et al. reported that CD8^+^ T cells didn’t indicate the anti-tumor immunity in NSCLC [21] and high infiltration of CD8^+^ T cells didn’t predict better prognosis. Our study also found that the proportion of CD8^+^ T cells was not associated with patient prognosis in NSCLC, but higher percentage of CD36^+^CD8^+^ T cells, as an independent factor, could predict both worse OS and RFS. This implied that in addition to distribution and number of CD8^+^ T cells, the state of CD8^+^ T cells was also important to induce anti-tumor immunity [22, 23].

We further analyzed the prognosis of patients who accepted postoperative chemotherapy at different stages and found that higher infiltration of CD36^+^CD8^+^ T cells was indicative of worse prognosis. This suggests that CD36 may lead to inferior response to chemotherapy in NSCLC patients by impairing anti-tumor immune functions. Targeting CD36 combined with chemotherapy is expected to bring better clinical benefits for NSCLC patients. Immune-checkpoint inhibitors (ICIs), particularly inhibitors of PD-1/PD-L1, have been confirmed great benefits in treatment of NSCLC [24–27], but there is still a considerable proportion of patients with little efficacy. Clinically, postoperative chemotherapy based on platinum is the first line therapy for most operable NSCLC patients. And combining ICIs with chemotherapy has been shown to improve survival in NSCLC patients [28, 29]. Nonetheless, many patients still respond poorly to this combination treatment. Therefore, the search for new immunotherapeutic targets is urgent. We attempted to block CD36 and detect changes in CD8^+^ T cell function, after employment of SSO, the cytotoxic function of CD36^+^CD8^+^ T cells was partially restored without altering the immune checkpoints, which indicated the potential of CD36 as a therapeutic target for NSCLC. Wang et al. [30] recently showed that immunotherapy-activated CD8^+^ T cells enhanced ferroptosis-specific lipid peroxidation in tumor cells, improving the anti-tumor efficacy of immunotherapy. CD36 was proved to mediate ferroptosis [10], and it seemed to contradict our aim of blocking CD36 to improve the anti-tumor immune response. However, through dual-color immunofluorescence, we found that CD36 was highly expressed on T cells rather than tumor cells in NSCLC (Fig. 2C and D), and CD36 blockage will exert little effect on the ferroptosis in tumor cells theoretically. Therefore, combination of other therapies including chemotherapy and CD36 blockage could promisingly enhance anti-tumor effects in NSCLC.

There were several limitations of our study. Firstly, we revealed the correlation of CD36^+^CD8^+^ T cells with TME and the prognosis of NSCLC; however, we didn’t explore the effect of CD36 alone, including how CD36 was regulated and whether there was a cause-and-effect connection between CD36 and TME or prognosis of NSCLC. And thus, a comprehensive understanding of CD36 in regulating TME and NSCLC progression is needed. Secondly, CD36 possibly had a greater prognostic impact on more extensive tumors (stage II-III) regardless of chemotherapy receipt. This indicated that it was not the best way to compare the prognosis (OS and RFS) among these patients who underwent surgery and adjuvant chemotherapy in either CD36^+^CD8^+^ T cells high or low group to explore the impact of CD36 on the response to chemotherapy. Instead, to really establish that CD36^+^CD8^+^ T cells impacted the immune response to chemotherapy specifically, the inferior response should be shown in patients with stage II-III NSCLC who had surgery and chemotherapy, and not shown in patients who had surgery alone. However, adjuvant postoperative chemotherapy is the standard of care in all patients at stage II-III [31], which means comparison can only be made in patients who underwent both surgery and chemotherapy. Thirdly, more explorations into mechanisms of CD36^+^CD8^+^ T cells inducing immunosuppressive TME in vitro and in vivo are needed. In addition, the effect and safety of combined CD36 blockage and chemotherapy need to be verified in animal models.

## Conclusions

The study found that high proportion of CD36^+^CD8^+^ T cells in the TME was an independent risk factor for poor prognosis in NSCLC patients, which predicted inferior chemotherapy response. CD36 blockage may enhance anti-tumor response and therapeutic effects of chemotherapy in NSCLC patients. However, more studies and prospective validations are needed to further explore the molecular mechanisms.

## Electronic supplementary material

Below is the link to the electronic supplementary material.

Supplementary Material 1

## Data Availability

All data generated that are relevant to the results presented in this article are included in this article and the supplementary materials. Other data that are not relevant for the results presented here are available from the corresponding author Dr. Jiang upon reasonable request.
