# Supplementary material for: Tumor-infiltrating CD36+CD8+T cells determine exhausted tumor microenvironment and correlate with inferior response to chemotherapy in non-small cell lung cancer
Source: BMC Cancer. 2023 Apr 21;23:367. doi: 10.1186/s12885-023-10836-z (PMC10120154; doi:10.1186/s12885-023-10836-z)
Supplement: Supplementary file 1 — Supplementary Material 1 [file 12885_2023_10836_MOESM1_ESM.docx]

**Supplementary table 1. Clinical characteristics of NSCLC patients**

| Characteristics | Patients | | |
| --- | --- | --- | --- |
|  | n |  | % |
| All patients | 232 |  | 100 |
| Age (y) |  |  |  |
| Mean ± SD |  | 57.2±9.2 |  |
| <60 | 128 |  | 55.2 |
| ≥60 | 104 |  | 44.8 |
| Gender |  |  |  |
| Female | 114 |  | 49.1 |
| Male | 118 |  | 50.9 |
| Smoke |  |  |  |
| Yes | 89 |  | 38.4 |
| No | 143 |  | 61.6 |
| EGFR Mutation |  |  |  |
| Yes | 126 |  | 54.3 |
| No | 106 |  | 45.7 |
| PD-L1 |  |  |  |
| High | 98 |  | 42.2 |
| Low | 134 |  | 51.8 |
| Tumor size (cm) |  |  |  |
| Mean ± SD |  | 2.7±1.3 |  |
| Pathological stage |  |  |  |
| I | 72 |  | 31.0 |
| IIA | 48 |  | 20.7 |
| IIB | 39 |  | 16.8 |
| III | 68 |  | 29.3 |
| IV | 5 |  | 2.2 |
| Lymph node metastasis |  |  |  |
| Yes | 94 |  | 40.5 |
| No | 138 |  | 59.5 |
| Histological type |  |  |  |
| Adeno- | 128 |  | 55.2 |
| Squamous- | 104 |  | 44.8 |
| Differentiation |  |  |  |
| Good | 147 |  | 63.4 |
| Poor | 85 |  | 36.6 |
| CD8^+^ T cells number |  |  |  |
| High | 121 |  | 52.2 |
| Low | 111 |  | 47.8 |
| CD36^+^CD8^+^ T cells number |  |  |  |
| High | 102 |  | 43.9 |
| Low | 130 |  | 56.1 |

**Supplementary table 2. Clinical characteristics of 53 NSCLC patients**

| Characteristics | Patients | | |
| --- | --- | --- | --- |
|  | n |  | % |
| All patients | 53 |  | 100 |
| Age (y) |  |  |  |
| Mean ± SD |  | 53.6±11.1 |  |
| <60 | 31 |  | 58.5 |
| ≥60 | 22 |  | 41.5 |
| Gender |  |  |  |
| Female | 30 |  | 56.6 |
| Male | 23 |  | 43.4 |
|  |  |  |  |
| Smoke |  |  |  |
| Yes | 18 |  | 40.0 |
| No | 35 |  | 60.0 |
| EGFR Mutation |  |  |  |
| Yes | 22 |  | 41.5 |
| No | 31 |  | 58.5 |
| Tumor size (cm) |  |  |  |
| Mean ± SD |  | 2.8±1.4 |  |
| Pathological stage |  |  |  |
| I | 10 |  | 18.9 |
| IIA | 11 |  | 20.8 |
| IIB | 6 |  | 11.3 |
| III | 25 |  | 47.2 |
| IV | 1 |  | 1.9 |
| Lymph node metastasis |  |  |  |
| Yes | 14 |  | 26.4 |
| No | 39 |  | 73.6 |
| Histological type |  |  |  |
| Adeno- | 29 |  | 54.7 |
| Squamous- | 24 |  | 45.3 |
| Differentiation |  |  |  |
| Good | 31 |  | 58.5 |
| Poor | 22 |  | 41.5 |

**Supplementary table 3. Immunohistochemistry (IHC) antibodies**

| No. | Antibody name | Clonality Species | Company | Product. No | Diluted | Identical cells |
| --- | --- | --- | --- | --- | --- | --- |
| 1 | Anti-CD8 alpha antibody | Monoclonal Rabbit Anti-human | Abcam | ab217344 | 1:400 | CD8^+^ T cells  CD36^+^ CD8^+^ T cells |
| 2 | Anti-CD36 antibody | Monoclonal Rabbit Anti-human | Abcam | ab252922 | 1:1000 |  |
| 3 | Anti-CD4 antibody | Monoclonal Rabbit Anti-human | Abcam | ab183685 | 1:150 | CD4^+^ T cells  Th1 (Type 1 helper T cells)  Th2 (Type 2 helper T cells) |
| 4 | Anti-T-bet antibody | Monoclonal Mouse Anti-human | Abcam | ab275959 | 1:500 |  |
| 5 | Anti-GATA3 antibody | Monoclonal Mouse Anti-human | Abcam | ab282110 | 1:200 |  |
| 6 | Anti-FOXP3 antibody | Monoclonal Mouse Anti-human | Abcam | ab20034 | 1:1000 | Tregs (T regulatory cells) |
| 7 | Anti-NCAM1 antibody | Monoclonal Rabbit Anti-human | Abcam | ab220360 | 1:1000 | NKs (Natural killer cells) |
| 8 | Anti-CD11c antibody | Monoclonal Rabbit Anti-human | Abcam | ab52632 | 1:500 | DCs (Dendritic cells) |
| 9 | Anti-CD86 antibody | Monoclonal Rabbit Anti-human | Abcam | ab239075 | 1:500 | M1-polarized macrophages |
| 10 | Anti-CD163 antibody | Monoclonal Rabbit Anti-human | Abcam | ab182422 | 1:100 | M2-polarized  macrophages |

**Supplementary table 4. Flow cytometry (FCM) antibodies**

| No. | Antibody name | Clonality Species | Company | Product. No | Dye | Marker |
| --- | --- | --- | --- | --- | --- | --- |
| 1 | BV421 Mouse Anti-Human CD45 | HI30 | BD Biosciences | 563879 | BV421 | CD45 |
| 2 | APC/Cy7 anti-human CD3 Antibody | SK7 | Biolegend | 344818 | APC/Cy7 | CD3 |
| 3 | BB515 Mouse Anti-Human CD8 | RPA-T8 | BD Biosciences | 564526 | BB515 | CD8 |
| 4 | PerCP/Cy5.5 anti-human CD36 Antibody | A1 | Biolegend | 328218 | PerCP/Cy5.5 | CD36 |
| 5 | PE Mouse anti-Human CD279(PD-1) | EH12.1 | BD Biosciences | 560795 | PE | PD-1 |
| 6 | Brilliant Violet 421anti-human TIGIT(VSTM3) | A15153G | Biolegend | 372710 | BV421 | TIGIT |
| 7 | Brilliant Violet 785 anti-human CD366 (Tim-3) | F38-2E2 | Biolegend | 345032 | BV785 | Tim-3 |
| 8 | Alexa Fluor 700 Mouse Anti-Human IFN-γ | B27 | BD Biosciences | 557995 | AF700 | IFN-γ |
| 9 | Alexa Fluor 700 anti-human/mouse Granzyme B | QA16A02 | Biolegend | 372222 | AF700 | GZMB |
| Intracellular cytokine stimulator | | | | | | |
| Phorbol-12-myristate-13-acetate (BD Biosciences 1:1000) | | | | | | |
| Ionomycin (BD Biosciences 1:1000) | | | | | | |
| Brefeldin A (BD Biosciences 1:500) | | | | | | |

**Supplementary Figure 1 Gating Strategy**

**
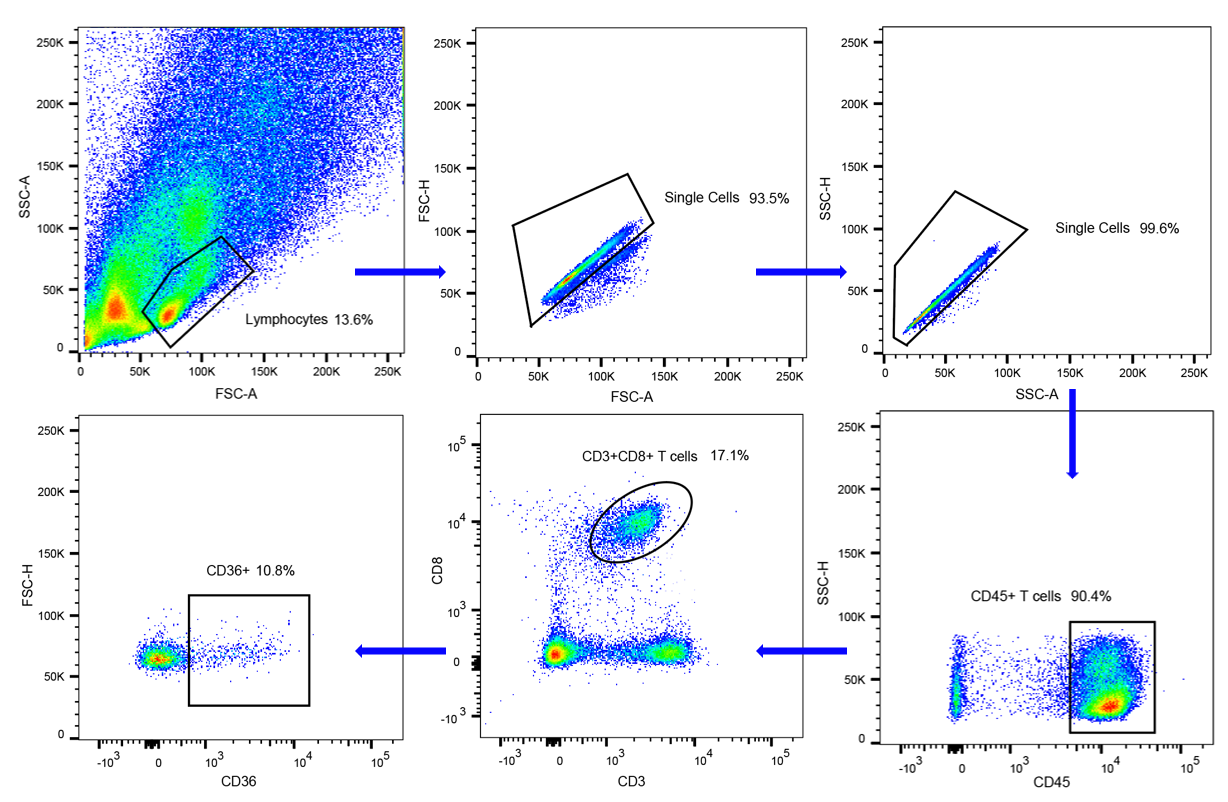
**
